# Colonies of Bumble Bees (*Bombus impatiens)* Produce Fewer Workers, Less Bee Biomass, and Have Smaller Mother Queens Following Fungicide Exposure

**DOI:** 10.3390/insects6020478

**Published:** 2015-06-01

**Authors:** Olivia M. Bernauer, Hannah R. Gaines-Day, Shawn A. Steffan

**Affiliations:** 1 Department of Entomology, University of Wisconsin, 1630 Linden Dr., Madison, WI 53706, USA; E-Mails: ombernauer@wisc.edu (O.M.B.); hgaines@wisc.edu (H.R.G.-D.); 2 United States Department of Agriculture, Agricultural Research Service, Vegetable Crops Research Unit, Madison, WI 53706, USA

**Keywords:** chlorothalonil, bee decline, native bees

## Abstract

Bees provide vital pollination services to the majority of flowering plants in both natural and agricultural systems. Unfortunately, both native and managed bee populations are experiencing declines, threatening the persistence of these plants and crops. Agricultural chemicals are one possible culprit contributing to bee declines. Even fungicides, generally considered safe for bees, have been shown to disrupt honey bee development and impair bumble bee behavior. Little is known, however, how fungicides may affect bumble bee colony growth. We conducted a controlled cage study to determine the effects of fungicide exposure on colonies of a native bumble bee species (*Bombus impatiens*). Colonies of *B. impatiens* were exposed to flowers treated with field-relevant levels of the fungicide chlorothalonil over the course of one month. Colony success was assessed by the number and biomass of larvae, pupae, and adult bumble bees. Bumble bee colonies exposed to fungicide produced fewer workers, lower total bee biomass, and had lighter mother queens than control colonies. Our results suggest that fungicides negatively affect the colony success of a native bumble bee species and that the use of fungicides during bloom has the potential to severely impact the success of native bumble bee populations foraging in agroecosystems.

## 1. Introduction

Bees are among the most important pollinators, providing vital services to natural and agricultural systems. Approximately 90% of flowering plants, including 35% of all crops, require insect mediated pollination [1,2]. Managed honey bees are the most widely used bees for crop pollination, although for certain crops, native bees are more effective pollinators than honey bees (e.g., cranberry, [3]; apple, [4]; cherry, [5]; alfalfa, [6]). Unfortunately, both managed honey bees and wild, native bees have been declining around the world [7,8,9,10]. Although the exact cause of these declines is uncertain, several factors have been implicated including habitat loss and fragmentation, disease, and exposure to agricultural chemicals [7,11,12]. Gaining a better understanding of each of the factors driving these declines is vital to ensure the continued provisioning of pollination services to both natural and agricultural systems.

Exposure to agricultural chemicals is one factor implicated in bee declines that has received much attention in both the scientific and popular media [13,14,15,16,17,18]. The majority of research focuses on insecticides (e.g., [19,20,21]); however, increasing evidence suggests that fungicides may also have detrimental effects on bees [22,23,24,25]. For example, honey bee colonies exposed to chlorothalonil, a commonly used, broad-spectrum fungicide, had higher rates of mortality than colonies not exposed to the chemical [24]. Furthermore, honey bee larvae reared on pollen contaminated with fungicides (Captan^®^, Rovral^®^, or Ziram^®^) failed to develop into adults [25]. Additionally, Pettis *et al*. [26] found that 100% of honey bee-collected pollen in agricultural landscapes contained fungicide residue. This suggests that fungicide exposure to honey bees is common and frequent. Since wild, native bees are also important crop pollinators, it is likely that they, too, run the risk of being exposed to fungicides if foraging within agricultural landscapes. It is critical, therefore, to examine the effects of fungicides on both managed and native bees.

Bumble bees are known to be important pollinators in many systems [1,27,28] and are unique among bees native to North America because they exist both as wild colonies as well as commercially available managed colonies, making them a model organism to study how agrochemical exposure affects wild bees. Of the few studies that have investigated the effect of fungicides on bumble bees, the results were conflicting (e.g., altered foraging behavior, [23], no effect [29,30] no effect on brood production yet repellency to adults [31]). Few studies, therefore, have documented the effect of fungicides on bumble bee colony growth and success. Here, we have conducted a controlled caging study to isolate the impacts of the fungicide, chlorothalonil, on the colony success of a North American bumble bee species (*Bombus impatiens* Cresson). Chlorothalonil is a widely used fungicide on crop and ornamental plants [32] and is commonly found in high concentrations within the pollen and hives of honey bees foraging in agricultural systems [14,26]. Well-known products that contain chlorothalonil as their active ingredient include Bravo^®^, Daconil^®^, and Sweep^®^ [33]. The initial intended use of chlorothalonil was for fungal diseases of turf grasses [34], but it was later approved for use on food crops and is now applied to a wide variety of flowering nut, vegetable, and fruit crops [33]. The application of chlorothalonil to flowering agricultural crops means that there is the potential for pollinators to come in contact with this compound. For example, Mullin *et al*. [14] surveyed honey bee colonies from 23 states and found that chlorothalonil was the most frequently detected pesticide in pollen and wax samples. Additionally, chlorothalonil has been found within “entombed pollen” in honey bee colonies that suffered from colony collapse disorder (CCD) [24]. The fact that honey bees are commonly exposed to chlorothalonil suggests that bumble bees and other native species foraging in agricultural crops are also being exposed. Given the negative impacts associated with chlorothalonil exposure in honey bees [24], we hypothesize that bumble bee colonies exposed to this fungicide will have reduced colony success, as measured by number and biomass of larvae, pupae, and adult bees.

## 2. Materials and Methods

### 2.1. Experimental Set-Up

To assess the effect of fungicide exposure on bumble bee colony success, we conducted a cage study at the West Madison Agricultural Research Station (Madison, WI). Ten mesh cages (3 m × 3 m base × 2 m tall, Ozark Trail Instant 10' × 10' Screen Houses) were set up in a field planted with oats (*Avena barbata*). A trench was dug around each cage and all four edges were buried into the ground to ensure that bees could not escape. Cages were stocked with potted, flowering plants known to be attractive to bees: buckwheat (*Fagopyrum esculentum*), borage (*Borago officinalis*), alyssum (*Lobularia maritima* cultivar), cosmos (*Cosmos bipinnatus* cultivars), and sunflowers (*Helianthus annuus* cultivars). Cages were additionally supplemented with a single tray (36 × 42 cm) of in-bloom clover (*Trifolium* sp.). Floral resources were clustered within one corner of the cage occupying a space approximately 2.5 m × 1 m. The remaining area of the cage was evenly vegetated by the oats. Bumble bee colonies (*Bombus impatiens*, research “mini-colonies,” Koppert Biological Systems, Howell, MI) each containing workers and a single queen, were randomly assigned a treatment (fungicide +/−, *n* = 5 colonies per treatment), then placed within a field cage (*n* = 1 colony per cage) for 29 days (23 June–21 July 2014). Initial colony weight did not differ between treatments (*F*_1,9_ = 0.18, *p* = 0.68). Colonies were retrieved from the field prior to producing gynes and placed in a freezer.

To protect the colony boxes from direct sun and rain, colonies were placed in plastic crates wrapped in insulated bubble wrap and set on two bricks. Crates were staked down with two large turf stakes and a piece of parachute cord. Colony boxes were oriented so that the colony’s opening was facing south to provide the bees with optimal navigational conditions [35]. Colonies were provided sugar water bladders to supplement nectar availability (similar to [17]), which remained in place for the duration of the study.

The fungicide chlorothalonil (Bravo^®^ with Weatherstik, Syngenta AG, Basel, Switzerland) was applied at a field-relevant level (20 g/L) to flowering plants in the five fungicide treatment cages using a hand held pesticide sprayer (RL FloMaster Yard and Garden Sprayer, 1 Gallon, Model 1401P) twice during the study (day 0 and 13). Fungicide was applied as “spray to drip,” meaning that all flowers were uniformly coated such that no further liquid could adhere to floral surfaces. Approximately 0.8 L of liquid was applied per cage. This corresponds to ~17 kg/ha of the active ingredient, chlorothalonil, which is below the upper range of allowable rates for ornamental plants and turf. Fungicide was only applied to plants. Colonies were present in the cages when the sprays occurred, but colonies were not sprayed directly. Applications were made in the late evening at and after dusk and on days with little or no wind or rain.

### 2.2. Sampling and Analyses

At the conclusion of the study, *B. impatiens* colonies were collected from the cages. Exit doors were blocked 24 h prior to collection, allowing bees to return to the colony box but not leave. Any bees that were seen outside the colony at the time of retrieval were collected into a vial and kept with their respective colony. The following response variables were measured: number and dry-weight of larvae, pupae, workers (*i.e.*, adult female foragers), adult males, and the mother queen.

Before analyzing the data, we used a Shapiro-Wilk Goodness of Fit test to assess whether the data met the assumptions of normality and 2-sided *F*-test to assess whether variances were equal. For variables that met the assumptions, a one-way ANOVA was performed with treatment as the independent variable. For data that did not meet the assumptions, a non-parametric Wilcoxon Rank Sum Test (also called Mann-Whitney *U* test) was performed. Statistics related to normality and variance are presented in Table 1. All statistical analyses were conducted in JMP Pro 11 [36].

## 3. Results

**Figure 1:**
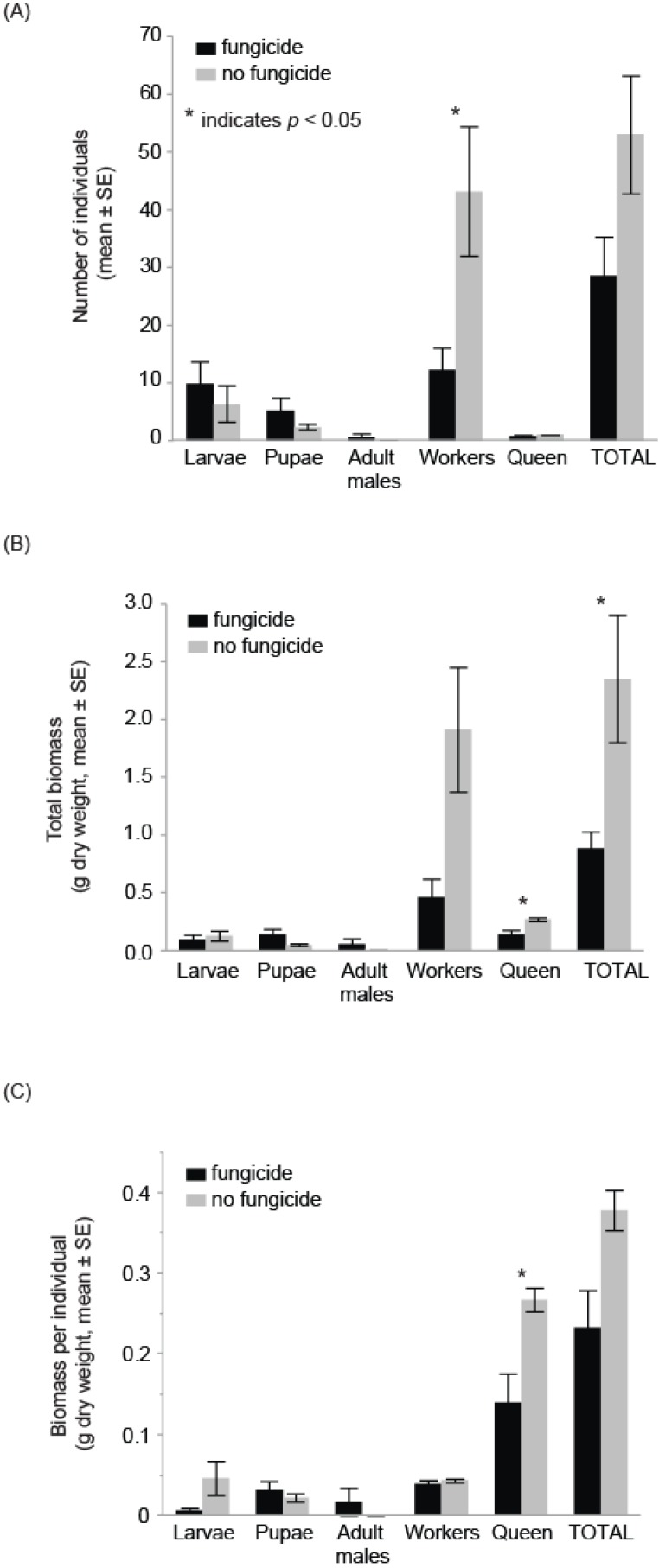
Bumble bee colonies exposed to the fungicide chlorothalonil had (**A**) fewer workers, (**B**) lower total bee biomass and (**C**) smaller mother queens than control colonies. Statistics for each life stage comparison can be found in Table 1.

Colonies exposed to the fungicide treatment (*n* = 5) produced less than a third as many workers (mean ± SE, fungicide 12.2 ± 3.8 *vs*. control 43.2 ± 11.2, *F*_1,9_ *=* 6.8, *p* = 0.03), less than half the amount of bee biomass (fungicide 0.91g ± 0.15 *vs*. control 2.36g ± 0.55, *F*_1,9_ = 8.3, *p* = 0.02) and had mother queens with half the body mass (fungicide 0.14g ± 0.04 *vs*. control 0.27 ± 0.01, *Z* = 2.5, *p* = 0.01) as compared to colonies in the no-fungicide control treatment (Table 1, Figure 1). The number of larvae, pupae, and males did not differ between treatments (Table 1). The biomass of each individual life stage (larvae, pupae, workers, and adult males) also did not differ (Table 1), nor did the weight per individual for larvae, pupae, and workers (Table 1). There were insufficient data to analyze the weight per individual for adult males by treatment.

## 4. Discussion

Over the past decade, declines among certain bee taxa have been documented worldwide [11]. One possible factor contributing to their decline is exposure to agricultural chemicals. Fungicides, used widely in agricultural systems, are generally considered safe for bees [29,31,37], but recent findings suggest otherwise (e.g., [22,23,25,30]). Using field cages, we found that bumble bee colonies exposed to the fungicide, chlorothalonil, resulted in significantly fewer worker bees, less total bee biomass and smaller mother queens than those in the control treatment.

One of the clearest patterns in our study was the significant reduction in the number of worker bees within the colonies exposed to fungicide. Colony success is dependent on the capacity of workers to collect resources and care for the queen, allowing the colony to grow and ultimately produce as many new queens as possible [38]. In order to maximize queen production, the colony must first collect sufficient resources to maximize forager numbers. With fewer foragers, a colony will be compromised in its capacity to support new queens. Thus, a colony that has a reduced number of foragers will likely have reduced fitness [38,39].

**Table 1:** Results of statistical analysis comparing the number, biomass, and biomass per individual for larvae, pupae, and adult (*i.e.*, female, male, and mother queen) bumble bees in fungicide treatments as compared to the non-fungicide control. For data that did not meet the assumptions of normality or equal variance, a non-parametric test was used.

*Y*-variable		Shapiro-Wilk †	*F*-test, 2-sided ‡	ANOVA	Wilcoxon Rank Sum
Number	Larvae	*W* = 0.92, *p* = 0.37	*F*_4,4_ = 1.5, *p* = 0.71	*F*_1,9_ = 0.47, *p* = 0.51	
	Pupae	*W* = 0.96, *p* = 0.84	*F*_4,4_ = 17.8, *p* = 0.02 *		*Z* = −0.74, *p* = 0.46
	Workers	*W* = 0.97, *p* = 0.92	*F*_4,4_ = 8.5, *p* = 0.06	*F*_1,9_ = 6.8, *p* = 0.03 *	
	Adult males	*W* = 0.62, *p* = 0.0001 ***	insufficient data		*Z* = −0.8, *p* = 0.42
	Total	*W* = 0.96, *p* = 0.80	*F*_4,4_ = 2.3, *p* = 0.43	*F*_1,9_ = 4.0, *p* = 0.08	
Biomass	Larvae	*W* = 0.95, *p* = 0.67	*F*_4,4_ = 1.1, *p* = 0.93	*F*_1,9_ = 0.27, *p* = 0.62	
	Pupae	*W* = 0.89, *p* = 0.17	*F*_4,4_ = 13.1, *p* = 0.03 *		*Z* = −1.5, *p* = 0.14
	Workers	*W* = 0.94, *p* = 0.58	*F*_4,4_ = 12.1, *p* = 0.03 *		*Z* = 1.7, *p* = 0.09
	Adult males	*W* = 0.62, *p* = 0.0001 ***	insufficient data		Z = −0.8, *p* = 0.42
	√(total)	*W* = 0.96, *p* = 0.79	*F*_4,4_ = 5.6, *p* = 0.12	*F*_1,9_ = 8.3, *p* = 0.02*	
Biomass per individual	Larva	*W* = 0.85, *p* = 0.08	*F*_4,4_ = 101.5, *p* = 0.003 **		*Z* = −1.1, *p* = 0.62
	Pupa	*W* = 0.94, *p* = 0.63	*F*_3,4_ = 3.9, *p* = 0.22	*F*_1,8_ = 2.2, *p* = 0.18	
	Workers	*W* = 0.96, *p* = 0.78	*F*_4,4_ = 5.1, *p* = 0.14	*F*_1,9_ = 0.80, *p* = 0.40	
	Adult male	insufficient data	insufficient data		insufficient data
	Queen	*W* = 0.81, *p* = 0.02 *	*F*_4,4_ = 6.2, *p* = 0.11		*Z* = 2.5, *p* = 0.01*

† *p* < 0.05 indicates that residuals are not normally distributed and transformation or use of non-parametric test is required; ‡ *p* < 0.05 indicates that variances are not the same and transformation or use of non-parametric test is required; * indicates *p* < 0.05; ** indicates *p* < 0.01; *** indicates *p* < 0.001.

Another pattern observed in our study was the significantly lower mother queen weight in colonies exposed to fungicides compared to the no-fungicide controls. Since the fungicide treated colonies also had fewer foragers, it is likely that the mother queens were not receiving sufficient food. Furthermore, a previous study has shown that fungicide exposure results in impaired bee foraging efficiency [23] suggesting that ostensibly unaffected workers may not be collecting adequate resources.

It should be noted that these patterns were observed within large field cages, an unnatural setting for a wild bee species. While this manipulation was critical to confine bee foraging to flowers with fungicide residue, we acknowledge that it likely introduced a degree of contrivance, and thus our results should be viewed in light of this. It should also be noted that any cage-effect was equally applicable to the colonies in the control cages, which were subjected to the same biotic and abiotic circumstances. Another limitation of our study was our relatively small sample sizes (five replications each of the fungicide/no-fungicide treatments). Greater sample sizes are often helpful to distinguish among treatments, particularly where variances are large, but our sample sizes provided adequate data to discern treatment effects. Another element of our study that might have been improved was the inclusion of bee-counts and/or measurement of total bee biomass prior to the start of the experiment. We did measure total colony biomass prior to the start of the study, but we did not specifically weigh or count the bees in each colony. Doing so may have compromised bee health to an unknown degree, so we opted to weigh the entire colony instead. No significant differences were found among any of the founding colonies. Finally, at the conclusion of our study we measured multiple response variables relevant to bee fitness but could not measure queen production because by mid-season, the colonies had not yet begun to produce new queens. However, measurement of mid-season forager abundance represents a closely linked proxy for queen productivity, given that colonies with more foragers are known to produce more queens [38,39].

While previous research suggests that fungicides are not lethal to adult bees [29,31,37], our results demonstrate that fungicide exposure results in smaller colony size in the native North American bumble bee, *Bombus impatiens*. These results suggest that a mechanism other than acute toxicity is responsible for lower colony success. There are many potential factors that may be driving these impacts on *B. impatiens*. One possible mechanism is a disruption of the microbial community associated with bees. Previous researchers have documented strong associations between bees and microbes, including bacteria and fungi, especially yeasts [40,41]. This research suggests that microbes play an important role in the health of bees by, for example, preventing pollen provisions from spoiling and providing added vitamins (e.g., yeasts produce critical B vitamins, [42]) to their diet. If pollen collected by bees is contaminated with fungicides, it stands to reason that the community of beneficial microorganisms, specifically fungi, may be altered, and the digestive and nutritional benefits provided by these microbes could be compromised. One study has already demonstrated changes in the fungal community in pollen collected by honey bees after exposure to fungicides [43] suggesting that similar patterns may be true for other bees. Future research should characterize how fungicide residues may be altering the microbiome of pollen-provisions, and thus the nutrition of native bees.

## 5. Conclusions

We provide empirical evidence that one particular fungicide, chlorothalonil, can cause significant colony losses in bumble bees. Specifically, we report that the number of worker bees in *Bombus impatiens* colonies was reduced from a mean of 43.2 bees in control cages to 12.2 in fungicide-treated cages. Such losses drove the significant reduction in total colony biomass, a consequence that directly affects the production of new queens in the fall [38,39]. Additionally, the mean weight of mother queens among the fungicide-treated colonies was significantly reduced from that of controls, indicating there were sub-lethal consequences of chlorothalonil. These findings call into question whether fungicides are completely bee-safe for all bee species, and point to the need for further studies examining the acute and chronic impacts of fungicides on native bees. The trade-offs associated with fungicide use during bloom will also likely need to be re-visited.
